# Reply

**Published:** 2010

**Authors:** Attia Z. Taha

**Affiliations:** Family and Community Medicine Department, King Faisal University Dammam, Saudi Arabia aztaha@hotmail.com

We would like to thank Dr. Aggarwal for his comments about the article. Dr. Aggarwal was right in stating that a greater percentage of students from the College of Dentistry were ‘shisha’ smokers as compared to other two groups. The misinterpretation could be due to the fact that we used a column total instead of row total as a denominator in Table 3. We realized this when constructing Table 3 (that a high prevalence of shisha use is seen among dental students and least among the medical students). However, our aim was to highlight the proportion of shisha smokers in each college in relation to the total number of students who smoke. However, the use of either column total or row total will not change the result of the statistical association. Yes, the identity of the students was concealed while questionnaires were filled. No names appeared on the questionnaires. This is an essential ethical consideration in conducting such a study.

